# Incidence of Vitamin D Insufficiency in Coastal South-Eastern US Patient Population With Cardiovascular Disease

**DOI:** 10.14740/jocmr1953w

**Published:** 2014-09-09

**Authors:** Sherrie Khadanga, Clara V. Massey

**Affiliations:** a Department of Medicine, University of Vermont, 111 Colchester Avenue, Fletcher 311, Burlington, VT, USA; b Division of Cardiology, University of South Alabama, Mobile, AL, USA

**Keywords:** Vitamin D insufficiency, Serum 25-hydroxyvitamin D, Cardiovascular disease, Body mass index

## Abstract

**Background:**

Vitamin D insufficiency is increasingly gaining prominence as an associated cardiovascular disease (CVD) risk factor, often thought to be an issue in colder climates and higher altitudes. The intent of this study was to ascertain vitamin D levels in the southern Alabama gulf-coast region that has a high number of sunny days along with an annual average elevated UV ray index.

**Methods:**

An observational retrospective study of 204 patients with established CVD treated at the University of South Alabama’s Heart Center from January 2007 through January 2013 was undertaken. One-way ANOVA analyses were performed to determine any significant difference in the mean 25-hydroxyvitamin D (25(OH)D) serum based on gender and also based on race/ethnicity. Further, odds ratio (OR) was computed to ascertain if there was a relationship between vitamin D insufficiency and elevated body mass index (BMI).

**Results:**

Out of 204 patients, 53.4% (n = 109) were found to have vitamin D insufficiency (25(OH)D = 20.1 ng/mL), while 46.6% (n = 95) were within the normal range (25(OH)D = 37.8 ng/mL). The mean 25(OH)D of the entire group was 28.3, indicating a general trend of vitamin D insufficiency for patients treated at the cardiology clinics.

**Conclusion:**

This study established the prevalence of vitamin D insufficiency in the hot and high UV ray index climate of the coastal south-eastern United States. Also, it revealed the relationship of increased BMI with low 25(OH)D serum level. More extensive studies should be conducted in similar climates to further assess vitamin D insufficiency.

## Introduction

Cardiovascular disease (CVD) risk factors are multifaceted. Factors like hypertension (HTN), chronic kidney disease, hyperlipidemia (total cholesterol, LDL, HDL, and triglycerides), diabetes mellitus (DM), peripheral artery disease (PAD), C-reactive protein, obesity, smoking, and sedentary lifestyle have been of primary focus of medical treatment in clinical settings. The issue of vitamin D deficiency is gaining importance for cardiovascular risk assessment. The medical community is gradually acknowledging the association of low levels of vitamin D with an increased risk of cardiovascular events and introducing vitamin D supplementation in clinical settings [1-4]. However, there seems to be a general assumption that vitamin D deficiency occurs mostly in colder climates and in higher altitudes [5].

Numerous studies pertaining to vitamin D have been undertaken both in the form of original research and review research. Vitamin D studies have been undertaken in the field of rheumatology [6-8], nephrology [9-11], orthopedics [12-14], and endocrinology [15-17] among others. While there are studies addressing vitamin D deficiency in cardiology [18-22], relatively few of them have been undertaken in a cardiovascular setting within a university medical center specifically addressing costal populations in a hot, sunny, and high UV index environment.

The intent of this study is to ascertain vitamin D levels among cardiac patients in the southern Alabama gulf-coast region (latitude N 30.69/longitude W 88.05) [23]. This region has 218 sunny days considered to be relatively high [24] along with an annual average elevated UV ray index between 7 and 8 [25]. Additionally, a “beach culture” prevails where outdoor activities, however minimal, are a part of routine life that provides sun exposure, whether planned or not. It is postulated that any findings of this study would be of significance not only to the immediate geographical proximity, but also to other areas with similar sun exposure and topography.

## Materials and Methods

An observational retrospective study of patients with established CVD (n = 204) was undertaken. The study was approved by the University of South Alabama’s Institutional Review Board (IRB). The patients were treated at the University of South Alabama College of Medicine (USACOM) Heart Center from January 2007 through January 2013.

The plasma metabolite of vitamin D, 25-hydroxyvitamin D (25(OH)D) has been thought by researchers to be a useful risk marker in studying vitamin D deficiency [26, 27]. The determination of vitamin D insufficiency for the purpose of this study has been designated as a value of 25(OH)D being < 30 ng/mL.

A patient chart review and pursuant data collection was done to obtain the following data points: gender, race, age, body mass index (BMI), and vitamin D serum level. BMI was calculated as weight (kg) divided by height (m^2^). BMI range was divided into two categories based on the common definitions of “normal” (< 25) and “overweight” (≥ 25). It needs to be noted that patients with vitamin D supplementation were excluded from the study; only those without any vitamin D supplementation were considered. Being actively treated in a cardiology clinic, most patients were on statins, anti-hypertension medications, and DM therapies. Since the distinct goal of this study was to determine the incidence of vitamin D insufficiency, other observation points (lipid profile, HTN data, and A1c) were excluded.

Statistical analyses were undertaken using SYSTAT 12.0 (Systat Software Inc., San Jose, CA, USA). Descriptive statistical analysis was conducted to present the baseline characteristics (age, gender, race/ethnicity, BMI, BMI range, and serum 25(OH)D ng/mL). Percentages were calculated for patients with vitamin D insufficiency, further categorizing them into “insufficiency” and “deficiency” based on gender and race/ethnicity. A cut off point of < 20 ng/mL and < 30 ng/mL was used for specifying vitamin D deficiency and insufficiency. Values are presented as mean ± standard deviation. One-way ANOVA analyses were performed to determine any significant difference in the mean 25(OH)D serum based on gender and also based on race/ethnicity. A P value of < 0.05 was considered to indicate statistical significance. Further, odds ratio (OR) was computed to ascertain any relationship between vitamin D insufficiency and elevated BMI.

## Results

Baseline characteristics of the total study population including patients with and without vitamin D insufficiency are presented in Table 1. The study included 80 (39.2%) male and 124 (60.8%) female patients. The average age for the entire population was 63.3 years with no significant mean age difference between male and female. Out of 204 patients, 53.4% (n = 109) were found to have vitamin D insufficiency (mean 25(OH)D = 20.1 ng/mL), while 46.6% (n = 95) were within the normal range (mean 25(OH)D = 37.8 ng/mL). What seemed to be remarkable was that the mean serum 25(OH)D of the total study population was 28.3, indicating a general trend of vitamin D insufficiency in patients treated at the cardiology clinic. It was observed that among African Americans, 74.3% (26 out of 35) had vitamin D insufficiency while 42.3% (79 out of 164) of whites had the same. Overall, 82.8% of the total population was overweight with a mean BMI of 29.5. Additionally, 86.2% of the population with vitamin D insufficiency had a higher BMI of 30.2 in comparison to population without vitamin D insufficiency.

**Table 1:** Baseline Characteristics

Variable	Total population (n = 204)	Population with 25(OH)D insufficiency (n = 109)	Population without 25(OH)D insufficiency (n = 95)
Age (years)	63.3 ± 11.9*	61.6 ± 13.1*	65.3 ± 10.1*
Gender			
Male	80 (39.2)†	42 (38.5)†	38 (40)†
Female	124 (60.8)	67 (61.5)	57 (60)
Race/ethnicity			
White	164 (80.4)	79 (72.5)	85 (89.5)
African American	35 (17.2)	26 (23.9)	9 (9.5)
Other	5 (2.4)	4 (3.7)	1 (1.1)
BMI range			
Normal (< 25)	35 (17.2)	15 (13.8)	20 (21.2)
Overweight (≥ 25)	169 (82.8)	94 (86.2)	75 (78.9)
BMI (kg/m^2^)	29.5 ± 5.4	30.2 ± 5.5	28.8 ± 5.4
Serum 25(OH)D (ng/mL)	28.3 ± 10.9	20.1 ± 6.0	37.8 ± 6.9

*Mean ± SD (all such values). †Data expressed as number (%).

Further analysis was undertaken to ascertain serum 25(OH)D insufficiency (< 30 ng/mL) and deficiency (< 20.0 ng/mL) based on gender and race/ethnicity (Table 2). With respect to gender, female patients were more likely to be vitamin D deficient. However, a one-way ANOVA analysis did not reveal any significant variation in the mean vitamin D serum level between male and female patients (P value = 0.16).

**Table 2:** Serum 25(OH)D Deficiency and Insufficiency Level by Gender and Race/Ethnicity

Variable	Mean vitamin D serum level	Deficiency (< 20 ng/mL)	Insufficiency (< 30 ng/mL)
Gender			
Male	21.4 ± 5.7*	15 (37.5)†	27 (64.3)†
Female	19.4 ± 5.9	32 (47.8)	35 (52.2)
Race/ethnicity			
White	20.5 ± 5.7	34 (43.0)	45 (57.0)
African American	18.3 ± 6.7	13 (50.0)	13 (50.0)
Other	22.2 ± 2.7	0 (0.0)	4 (100.0)
Gender & race/ethnicity			
White male	21.7 ± 5.8	10 (31.2)	22 (68.8)
White female	19.8 ± 5.7	24 (51.1)	23 (48.9)
African American male	18.5 ± 6.5	5 (55.6)	4 (44.4)
African American female	18.2 ± 7.1	8 (47.1)	9 (52.9)

*Mean ± SD (all such values). †Data expressed as number (%).

In regard to race/ethnicity, white patients were more likely to have vitamin D insufficiency than deficiency. Percentage of patients having vitamin D deficiency and insufficiency was quite similar among African Americans. The analysis revealed that white male patients had more vitamin D insufficiency (68.8%) followed by African American female patients (52.9%) whereas African American male patients had more vitamin D deficiency (55.6%) followed by white female patients (51.1%). No significant difference in the mean serum 25(OH)D level was found between white and African American patients based on one-way ANOVA analysis (P value = 0.09).

**Figure 1:**
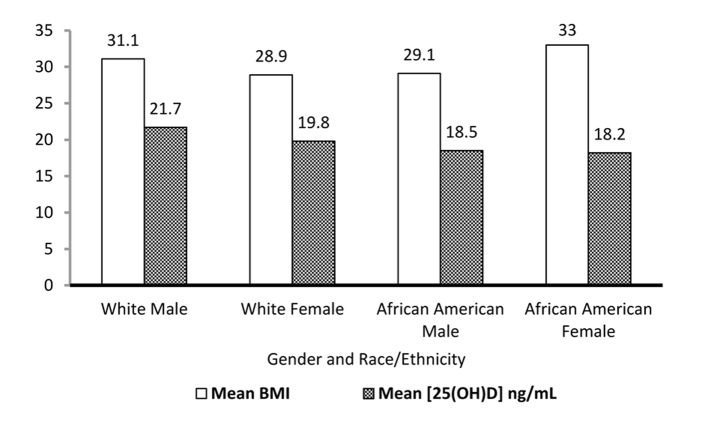
Mean serum 25(OH)D concentration and BMI of population with vitamin D insufficiency categorized by gender and race/ethnicity.

The comparison of mean serum 25(OH)D and mean BMI for population with vitamin D insufficiency based on gender and race/ethnicity is shown in Figure 1. Though their cardiac manifestations were wide-ranging that included atrial fibrillation, left ventricular hypertrophy, coronary artery disease, PAD, stable and unstable angina, it was apparent that the mean vitamin D serum level was relatively low across the broad spectrum of CVD. African Americans, especially women had both high mean BMI and low serum 25(OH)D level. The calculated OR (1.67) revealed that patients with a higher BMI (≥ 25) were more likely to have vitamin D insufficiency.

## Discussion

Vitamin D deficiency and the risk of CVD has been well established in previous studies [28-31]. The Framingham Offspring Study suggested that moderate to severe vitamin D deficiency is a risk factor for developing CVD [32]. The Copenhagen City Heart Study observed stepwise increases in the risk of heart disease as serum 25(OH)D level declined [33]. Ecological studies have reported that higher rates of CVD with increasing distance from the equator often attributed to the higher prevalence of vitamin D deficiency in colder climates and less exposure to sunlight [34-36]. Our study however focused on vitamin D insufficiency and the Southern Alabama gulf-coast region that has a high average temperature along with a high number of sunny days and an elevated UV ray index. The study substantiated the fact that vitamin D insufficiency levels exist in the coastal south-eastern region of the United States in spite of the high annual number of sunny days, and elevated UV ray index. In the University cardiovascular clinic setting, 53.4% of patients were found to have vitamin D insufficiency (< 30 ng/mL). Further, patients with a higher BMI were more likely to have lower Vitamin D serum levels. It is believed that these findings would be of relevance in clinical practice in the south-eastern region and other geographic areas with similar temperature, UV ray index, and topography. It is of interest to note that another study undertaken in Hawaii found that 51% of the population studied (n = 93) had low Vitamin D status using a cut point of 30 ng/mL [37]. Our study’s limitation is the relatively small sample size of 204 patients. This limitation may be somewhat ameliorated by the fact that all patients studied were receiving active treatment in the University Cardiology clinic and thereby of specific relevance to the field of cardiology.

### Conclusion

This study provides a perspective that vitamin D insufficiency levels are simply not limited to colder climates and higher altitudes. Further, patients with a higher BMI had an increased risk of vitamin D insufficiency. More large scale and extensive studies should be conducted in similar climates to further assess vitamin D insufficiency in cardiac patients to help establish guidelines for clinical practice.
